# EHMTI-0149. Low CSF hypocretin-1 levels are associated with cluster headache

**DOI:** 10.1186/1129-2377-15-S1-E14

**Published:** 2014-09-18

**Authors:** M Barloese, P Jennum, N Lund, S Knudsen, S Gammeltoft, R Jensen

**Affiliations:** 1 Neurology, Danish Headache Center, Glostrup, Denmark; 2 Neurology, Danish Center for Sleep Medicine, Glostrup, Denmark; 3 Diagnostics, Clinical Biochemistry Section, Glostrup, Denmark

## Background

Cluster headache (CH) is a debilitating headache disorder with strong chronobiological features characterized by severe pain attacks often waking the patient from sleep. Hypocretin (HCRT) is involved in the regulation of arousal and pain and is produced in the hypothalamus. HCRT has been suggested to play a role in the pathology of CH.

## Aim

To investigate HCRT-1 levels in CH during active bout.

## Methods

CSF HCRT was measured in 12 chronic and 14 episodic CH patients during active bout, and in 27 healthy controls. Patients were characterized beyond the dichotomy of current diagnostic criteria (episodic/chronic) using a CH index reflecting total headache duration and their attack rhythmicity compared to HCRT concentrations.

## Results

We found lower HCRT-1 levels in both chronic (388.67 pg/ml, p=0.0221) and episodic cluster headache (375.36 pg/ml, p=0.0005) compared with controls (430.96 pg/ml) and a tendency towards relatively higher values in chronic CH compared with episodic CH. A positive relationship between the CH index and HCRT concentrations was found in all patients (R2=0.1541, p=0.0473). We also identified a tendency towards relatively higher HCRT concentrations in patients without chronological rhythmicity.

## Conclusion

This is the first report of lower HCRT-1 concentrations in CH suggesting that decreased levels may reflect an insufficient anti-nociceptive activity of the hypothalamus. The exact mechanism of the anti-nociceptive effect of HCRT is not known and requires further investigation. In conclusion, this study supports the hypothesis of a connection between arousal- and pain- regulation and the pathogenesis of CH.

No conflict of interest.

